# Surgical correction in orbitotemporal neurofibromatosis with dystopia

**DOI:** 10.1186/s12886-016-0181-9

**Published:** 2016-01-07

**Authors:** Matthew Seung Suk Choi, Seung Hyup Choi, Jang Hyun Lee

**Affiliations:** Department of Plastic and Reconstructive Surgery, Hanyang University Guri Hospital, Hanyang University College of Medicine, 153 Gyeongchun-ro, Guri, 11923 South Korea

**Keywords:** Orbital dystopia, Neurofibromatosis, Ptosis

## Abstract

**Background:**

Orbitotemporal neurofibromatosis is a challenging disease for orbital surgeons. Ptosis correction may be needed following correction of orbital dystopia.

**Case presentation:**

A 34-year-old man, who underwent excision of a neurofibroma on the right eyelid in our clinic, returned to our clinic four years later complaining of dystopia and bulkiness of the protruding mass in the right eyelid and eyebrow. Computed tomographic imaging showed dysplasia and deformity in the sphenoid bone and orbit. A large mass was found in the superior portion of the orbit, protruding towards the temporal lobe, which in turn displaced the orbit downwards. A bicoronary incision and transcranial approach were performed, followed by the excision of the superior orbital space and temporal lobe mass by uncovering certain portions of the frontal, temporal, and zygomatic bones. After the excision of the mass, a calvarial bone graft was used to remodel the longitudinal widened orbit to correct the dystopia. While primary surgery was successful in the correction of dystopia, secondary surgery was performed to correct the exacerbated ptosis by levator muscle resection.

**Conclusions:**

Correction of orbitotemporal neurofibromatosis with dystopia involves three steps: removal of the mass in the orbit to eliminate the effect of downward dislocation of the orbit, placement of a bone graft in the orbit floor after repositioning the orbit for suspension and remodeling of the orbit, and following the correction of dystopia, ptosis may be corrected if needed.

## Background

Craniofacial neurofibromatosis can lead to not only soft tissue deformity but also bone deformity such as skull and facial bone deformity. Therefore, surgical treatment must focus on not only removing the mass but also correcting the deformed bony structure by remodeling to achieve facial symmetry [1, 2]. Among cases of craniofacial neurofibromatosis, half of the cases present with orbit and eyeball involvement, which makes correction even more difficult [1, 2]. There are two methods of correction of orbital dystopia: Orbital translocation is performed by subperiosteal exposure of the cranio-orbital skeleton and applying osteotomies near the orbit, whereas, orbital remodeling is performed by placing bone grafts and metallic plates in the orbit for reconstruction [3–5].

We used a bicoronal transcranial approach for resection of the mass in the superior orbit followed by zygomatic osteotomies for bone graft and reconstruction of the lateral wall and floor of the orbit for remodeling the orbit. Exacerbation of ptosis was significant despite the successful correction of orbital dystopia. Secondary surgery for ptosis correction was needed for a better cosmetic and functional result. Therefore, we report the sequence of an operative treatment method with a successful outcome for orbital neurofibromatosis and aggravated ptosis.

## Case presentation

A 30-year-old man visited our clinic in 2010 with an asymmetric orbit, right eye set 1 cm lower than the left and a soft, movable mass of 3 cm × 1 cm in his right eyelid, which all first appeared at the age of five. He had no signs of visual disturbance, including diplopia or limitation of the motion of the extraocular muscle. We planned to resect the eyebrow and eyelid mass and correct the dystopia; however, the patient refused to undergo orbital correction, so we settled for the excision of the eyelid mass. The mass was partially resected and was pathologically confirmed as neurofibroma.

**Fig. 1:**
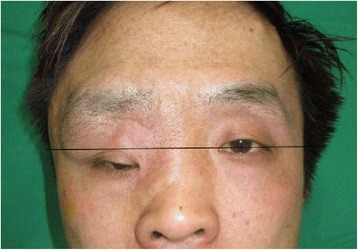
The protruding recurred mass in the right eyelid and eyebrow with orbital dystopia. Right eye set 1 cm lower than the left (*black line*)

**Fig. 2:**
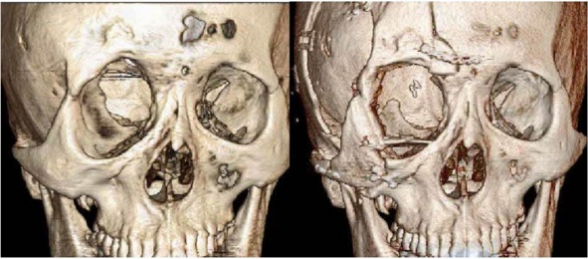
(*Left*) Pre-operative facial computer tomography of a 34-year-old man with orbitotemporal neurofibromatosis type 1. (*Right*) Post-operative facial computer tomography with L-shaped calvarial bone graft

Four years later, in March 2014, he returned to our clinic complaining about the protruding recurred mass in the right eyelid and eyebrow with orbital dystopia (Fig. 1). Facial computed tomography showed dysplasia of the right frontosphenoid and orbital bone, enlargement of the volume of the orbit, widening of the superior orbital fissure, and a mass located in the superior orbital space, protruding to the right temporal lobe. The mass effect of the superior orbital mass and the longitudinal widening of the orbit were presumed to be the reasons for the dystopia. Therefore, we planned to excise the mass and remodel the longitudinally widened orbit. A two-team approach with the neurosurgeons was adopted. Using a bicoronal incision, we exposed the frontal, temporal, and zygomatic bones. The neurofibroma mass in the supraorbital region was totally excised by opening the orbit roof and lateral wall by a transcranial approach and zygomatic osteotomies. After removing the mass, a 5 cm × 7 cm bone graft was harvested from the outer cortex of the bone fragment of the calvarium, which was excised when the craniectomy was performed. The bone graft was designed into an L-shape to restore the lateral wall and floor of the widened orbit (Fig. 2).

**Fig. 3:**
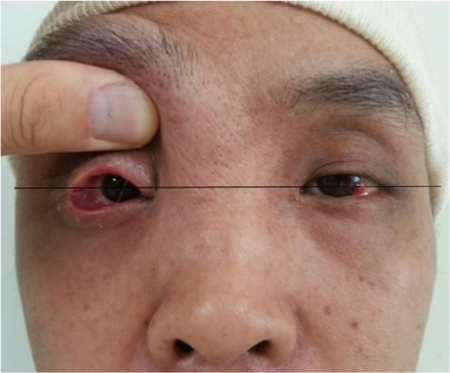
Clinical photograph after mass removal and orbital remodeling. Both eyes are located almost at the same level (*black line*)

**Fig. 4:**
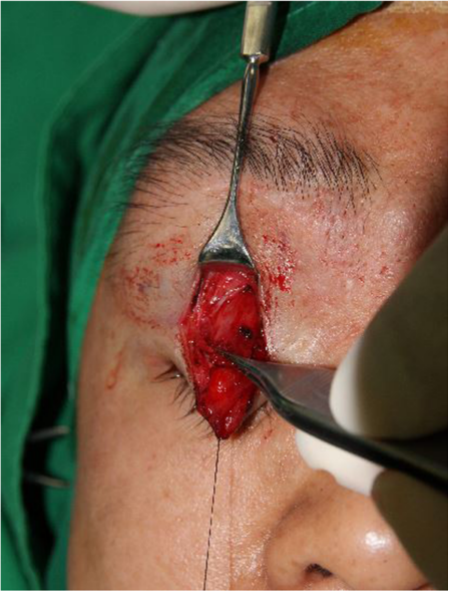
Clinical photograph of ptosis correction. A levator aponeurosis-Muller muscle composite flap can be seen

**Fig. 5:**
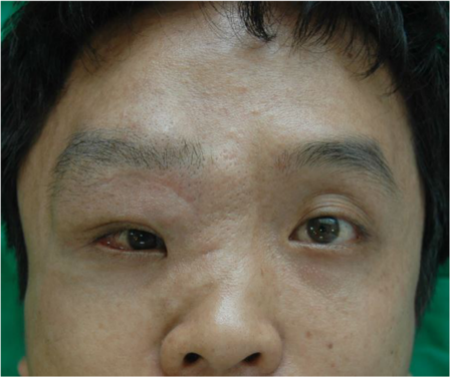
The final post-operative clinical photograph 4 months after ptosis surgery. Dystopia and ptosis were corrected

The right eyeball was elevated 1 cm upward by a calvarial bone graft in the orbit, resulting in both eyes being placed almost at the same level, restoring orbital symmetry (Fig. 3). However, as a result of this dystopia correction, ptosis was exacerbated from moderate to severe, with the degree of ptosis measuring 4 mm, and levator muscle function measuring 1 mm. Three months after the primary surgery, the periocular swelling had subsided, and the tissue was suitable for the secondary operation of ptosis correction. The Muller muscle and levator aponeurosis composite flap was elevated (Fig. 4) and fixed to the superior portion of the tarsal plate at three points. The patient was satisfied with the symmetric orbit and ptosis correction (Fig. 5).

## Discussion

Neurofibromatosis is an autosomal dominant trait with complete penetrance. Its frequency is one in 3000 live births. Skull and facial deformities occur in 22 % of cases. Malignant change may occur in 2 %–27 % of patients [1]. The treatment of orbitotemporal neurofibromatosis is challenging. The mass may be located near the orbit, which can be difficult to remove and may even cause dystopia. Exophthalmos with lowering of the globe and eyelid enlargement are the usual consequences of orbitopalpebral neurofibromatosis [6]. The surgical planning must include the evaluation of the exact amount of resection of the soft tissue and the requirement for bony involvement, and maintaining visual acuity as well as achieving a better aesthetic appearance [7]. If the affected eye has limited eyesight, it is possible to pay more attention to cosmesis; however, our patient had no visual disturbance.

Orbitotemporal neurofibromatosis was classified by Jackson into three groups according to the severity of the deformities: Group 1 (orbital soft tissue involvement with a seeing eye), Group 2 (orbital soft tissue and significant bony involvement with a seeing eye), and Group 3 (orbital soft tissue and significant bony involvement with a blind or absent eye) [4]. Our patient was classified as Group 2, since he had soft tissue and bony involvement with normal vision. The treatment in Group 2 involves an intracranial approach for tumor debulking and orbital wall reconstruction for narrowing the widened orbit with calvarial bone grafting. In our case, the tumor was situated in the superior orbital region, which pushed the eyeball downwards and widened the orbit. But there was no orbital wall defect. So we planned to resect the whole mass in order to gain space for the eyeball to be repositioned upwards. Then we placed a calvarial bone graft in the floor of the orbit to sustain the new position of the eyeball.

According to Morax et al. 7 % of patients with neurofibromatosis type 1 show skeletal changes of partial or complete absence of the greater wing of sphenoid bone [8]. This may lead to an enlargement of the superior orbital fissure, which results in herniation of the temporal lobe into the orbit, causing exophthalmos and ocular dystopia [9–12]. A skeletal change was present in our patient, who was treated for vertical orbital dystopia; however, superior orbital fissure repair was not performed during surgery since exophthalmos was not significant in our patient [13].

Blepharoptosis surgery in neurofibromatosis is indicated if the visual axis is compromised or if there is a chance of amblyopia. Correction of ptosis may be done primarily if it is the only problem, but should be delayed if there are other procedures to be done [7]. In our case, eyelid ptosis was aggravated after the orbital remodeling surgery, obstructing the visual field, so a secondary operation was performed after 3 months to correct the ptosis by levator muscle resection [14–16].

The timing of surgery in orbitotemporal neurofibromatosis is still controversial. Although a rapid growth phase of the neurofibromatosis is known to occur during childhood and puberty, most surgeons recommend undergoing surgery for orbitotemporal neurofibromatosis during early childhood, which may prevent orbital deformity and preserve eye function. Especially in cases of ptosis and pulsating exophthalmos, early surgery should be performed [7]. In contrast, correction of ptosis caused by an orbitotemporal mass for cosmetic purposes is delayed until the age of 18, when the disease progression has stabilized [17]. The recurrence rate of neurofibromatosis is relatively high. Ptosis of the upper eyelids may recur due to recurrence of the benign tumor. Recurrence may cause recurrence of exophthalmos as well [7]. In addition, the possibility of malignant change should not be overlooked. Therefore, long-term follow-up is needed to observe the consequences of the surgical treatment.

## Conclusions

Surgical treatment of orbitotemporal neurofibromatosis with vertical orbital dystopia begins by removing the mass in the supraorbital region to secure space for the upward correction of the depressed eyeball, followed by orbital remodeling by bone graft for repositioning the eyeball. We need to keep in mind that worsened ptosis may also occur after the correction of the vertical orbital dystopia, which may require a secondary ptosis correction.

## Consent

Written informed consent was obtained from the patient for publication of this case and any accompanying images. A copy of written consent is available for review by the Executive Editor of this journal.
